# Motif mining based on network space compression

**DOI:** 10.1186/s13040-014-0029-x

**Published:** 2014-12-11

**Authors:** Qiang Zhang, Yuan Xu

**Affiliations:** Key Laboratory of Advanced Design and Intelligent Computing, (Dalian university), Ministry of Education, Dalian, 116622 China

**Keywords:** Associated matrix, Space compression, Sub-graph mark, Parity nodes

## Abstract

A network motif is a recurring subnetwork within a network, and it takes on certain functions in practical biological macromolecule applications. Previous algorithms have focused on the computational efficiency of network motif detection, but some problems in storage space and searching time manifested during earlier studies. The considerable computational and spacial complexity also presents a significant challenge. In this paper, we provide a new approach for motif mining based on compressing the searching space. According to the characteristic of the parity nodes, we cut down the searching space and storage space in real graphs and random graphs, thereby reducing the computational cost of verifying the isomorphism of sub-graphs. We obtain a new network with smaller size after removing parity nodes and the “repeated edges” connected with the parity nodes. Random graph structure and sub-graph searching are based on the *Back Tracking Method*; all sub-graphs can be searched for by adding edges progressively. Experimental results show that this algorithm has higher speed and better stability than its alternatives.

**Electronic supplementary material:**

The online version of this article (doi:10.1186/s13040-014-0029-x) contains supplementary material, which is available to authorized users.

## Background

Researchers have discovered that the human genome is a complex network system. With the Human Genome Project (*HGP*), we step into a post-genome era. The network motif [1],[2] is generally represented as the smallest unit in a network. Motif analysis is increasingly recognized as a powerful approach to identify the function structure of a network, its organizational principle and species evolution.

The network motif was first defined systematically in Escherichia coli. After this, algorithms regarding network motif mining were developed. *Kashtan*, *Itzkovita, Milo* and *Alon*[3] proposed the Edge Sampling Algorithm (*ESA*), which unfortunately leads to sampling deviation and causes error. *Wernicke*[4] described an algorithm for enumerating sub-graphs, named Enumerating Subgraph *(ESU for short)*, which allows for a faster detection of network motifs and offers useful additional features. Considerable work has been made concerning subgraph searching, and we select the RAND-ESU method for its performance and unbiased estimation [5],[6]. Meanwhile, the generating random network model is a key to identifying motifs. We adopt the standard null model [7],[8], in which the degree of every node is not allowed to change, such that single node properties are fixed. *Lau* and *So*. [9] proposed the Markov Chain Algorithm to generate random networks [10], and we use it to construct the random networks for this paper. In addition, sub-graphs are another key to finding motifs because sub-graph isomorphism is an NP-complete problem [11],[12]. In order to solve this problem, a method aimed at reducing the searching size was introduced by *Ding* and *Huang*[13]. An algorithm to reduce the complexity of matching two graphs was proposed by *Knossow*,*Sharma, Mateus* and *Horaud*[14]. Another algorithm that optimizes the one-to-many matching problem was introduced by *Ogras* and *Marculescu*[15]. Moreover, some more recent algorithms have been developed [16], based on the G-trie method to list all stored subgraphs and the implicit tree method [17] to finding motifs of a size greater than eight. *Liao and Chen*[18] presented the Depth-First Spelling algorithm for mining sequential patterns of biological sequences with Gap constraints (termed DFSG). Zhang and Lu [19] employed a network stratification strategy to investigate the validity of the current network analysis of conglomerate PPI networks, finding that network stratification may help to resolve many controversies in the current research of systems biology. Srinivasan,*Vural, King* and *Guda*[20] presented a new substitution-based scoring function for identifying discriminative lower denominations that are highly specific to a class. Unfortunately, some of these methods, designed for both directed and undirected graphs, proved to be time-consuming. The aim of this paper is to achieve a method for reducing the searching time storage space required for a motif mining algorithm, while storing all sub-graphs.

We enumerate all sub-graphs that meet the requirements of a given graph by way of reducing the searching space. Then, the associated matrices of these sub-graphs are normalized and the isomorphic sub-graphs are uniquely marked. Through experiment, we verify the accuracy and extensive applicability of our algorithm, and improve the searching speed of sub-graphs enumeration.

In the following, we give some definitions:

### Definition 1

The *Network motif* is generally represented as a topological pattern that occurs more often in a given network than in random networks, and takes on a certain function in practical biological applications. We utilize graph theory to research network. A sub-graph is considered as a *Network Motif* when the following conditions are met [1],[2]:

1. The frequency *P* < 0.01, from 1000 randomized networks.
2. The number of sub-graph in the input graph *N*_real_ is larger than four.
3. Set*N*_real_ as the number of sub-graphs in the input graph, *N*_rand_ as the mathematical expectation of the random graphs, the following inequality should be satisfied: *N*_*real*_ − *N*_*rand*_ > 0.1*Nrand*.

### Definition 2

A *Network* is considered to be a large graph consisting of vertices and edges. A directed graph (or network) is usually indicated as *G* = (*V*, *E*), where *V* stands for a finite set of nodes in the graph and *E* the edges. Edge *e* = (*u*, *v*) ⊆ *E* represents an edge starting from the node *u* (the source) to the node *v* (the target).

### Definition 3

In a graph *G* = (*V*, *E*), any two nodes *V*_*i*_ and *V*_*j*_ that meet the following requirement are called *Parity nodes*[21].

i) Both in-degree and out-degree of *V*_*i*_ and *V*_*j*_ are the same, stated as $<d_{\mathrm{in}}^{i},d_{\mathrm{out}}^{i}>=<d_{\mathrm{in}}^{j},d_{\mathrm{out}}^{j}>$

### Definition 4

The frequency partition of a graph (simple graph) is a partition of its vertices grouped by their degree [22].

### Definition 5

Two sub-graphs *G* = (*V*_1_, *E*_1_) and *G*^/^(*V*_2_, *E*_2_) are *Sub-graph isomorphic*[12] if there is a one-to-one correspondence between their vertices, stated as *f*: *v*_1_ → *v*_2_, and also there is an edge correspondence *g*: *e*_1_ → *e*_2_, directed from one node to another node within one sub-graph. *e*_1_ ∈ *E*_1_, *Ψ*_1_(*e*) = < *u*, *v* > if and only if there is another edge with the same direction between the corresponding vertices in the other sub-graph *Ψ*_2_(*g*(*e*)) = < *f*(*u*), *f*(*v*) > .

**Figure 1:**
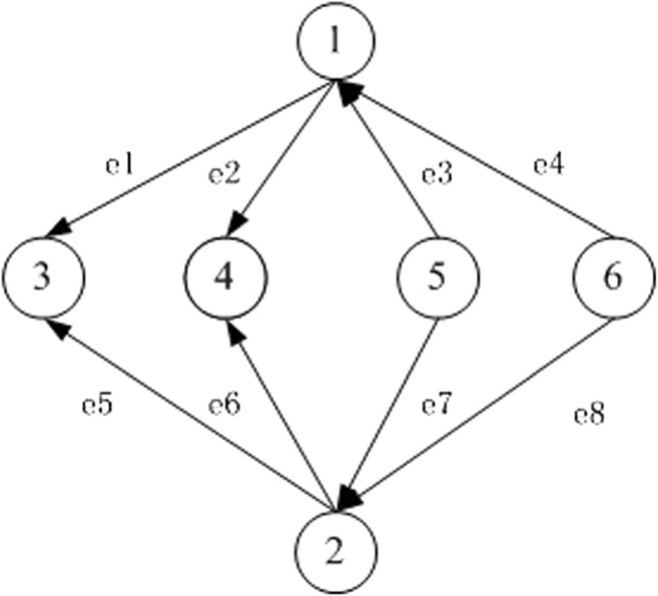
**An example of parity nodes.**

**Figure 2:**
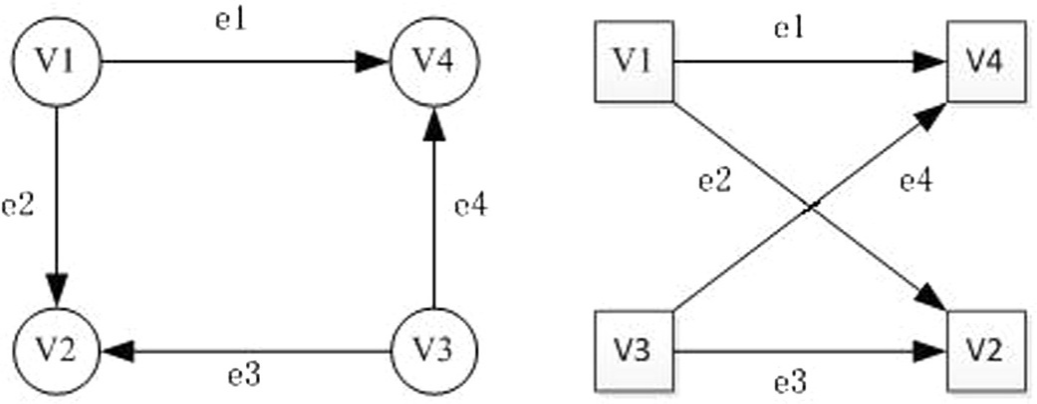
**An illustration of isomorphic graphs.**

Figure 1 is an example of parity nodes and Figure 2 shows the isomorphism visually.

## Methods

In this paper, we provide a new approach for motif mining based on compressing the searching space. Firstly, we use the associated matrices to store directed graphs, and then utilize parity nodes to compress searching space. Secondly, we use Back Tracking to enumerate all sub-graphs of a given size that occur in the input graph which are obtained from real graphs and random graphs. Thirdly, we normalize the associated matrix and mark isomorphism sub-graphs uniquely, by using the Symmetric Ternary to simulate the elements (−1, 0,1) in the associated matrix. And finally, we distinguish motifs among all the sub-graphs found on the basis of statistical parameters to identify it whether or not.

### Store graphs

The storage of graphs is the first step in the process of solving the motif-mining problem. The associated matrix is easy to compress and thus it is easy to know the size of a sub-graph, so we use the associated matrix to store real graphs and random graphs.

**Figure 3:**
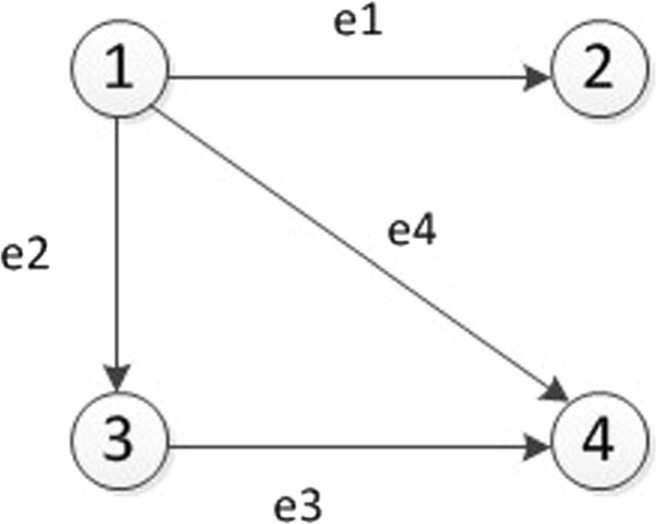
**An example of an associate matrix, showing the corresponding relationship between a graph and a form of storage.**

An associated matrix *M*(*G*) shows the connection between the nodes and edges of *G*. It is usually not a square matrix, shown as Figure 3

$$M\left(G\right)=\begin{array}{lllll} e_{1} & e_{2} & e_{3} & e_{4}\\ v_{1} & 1 & 1 & 0 & 1\\ V_{2} & -1 & 0 & 0 & 0\\ v_{3} & 0 & -1 & 1 & 1\\ v_{4} & 0 & 0 & -1 & -1 \end{array}$$

In this matrix, a row represents nodes, a column edges, and the algebraic sum of all elements is zero in *M*(*G*). In the matrix *M*(*G*), the sum of out-degree and in-degree are both equal to the number of edges, stated as $\sum_{i=1}^{n}\sum_{j=1}^{m}\left(m_{ij}=1\right)=m=-\sum_{i=1}^{n}\sum_{j=1}^{m}\left(m_{ij}=-1\right)$.

### Sub-graph mark and isomorphism

As is well known, the sub-graph isomorphism is an NP-complete [12] problem among the different types of graph matching algorithms. The required time increases exponentially with the size of the input sub-graphs. For an N-vertex sub-graph, the number of the node label permutation is N!. In addition, there is a difficult problem using the associated matrix to store graphs, because an element represents both the connection relationship and orientation directly, such as “-1”. Therefore, we need to adopt the concept of symmetrical three hexadecimal to extracting the matrix row by row for get sequence of symmetric ternary, and the symmetric ternary sequence is a Code number that is unique for each isomorphic class. In order to mark each graph uniquely, we need to standardize the associated matrix, which is useful for sub-graph isomorphism as it can reduce the complexity of sub-graph isomorphism and improve the efficiency of the searching. The detailed process of the standardization of the associated matrix can be referred to as [23].

$$\begin{array}{ccccc} e1 & e2 & e3 & e4\\ v1 & 1 & 0 & 0 & -1\\ v2 & 0 & -1 & 1 & 0\\ v3 & -1 & 1 & 0 & 0\\ v4 & 0 & 0 & -1 & 1 \end{array}\Leftrightarrow \begin{array}{ccccc} e1 & e2 & e3 & e4\\ v1 & 1 & 0 & 0 & -1\\ v3 & -1 & 1 & 0 & 0\\ v2 & 0 & -1 & 1 & 0\\ v4 & 0 & 0 & -1 & 1 \end{array}\Leftrightarrow {\left(100‐1‐11000‐11000‐11\right)}_{\mathrm{Symmetric}\;\mathrm{Ternary}}=13698880$$

From this example, we know that after two similar matrices get elementarily transformed; we can get the same matrix and a unique Code. One Code marks an isomorphic class. We just have to count the same values of the Codes to detect the motif. We confirm the sufficiency and necessity of this unique mark theoretically, this being outlined below:

#### The sufficiency

If isomorphism exists, their associated matrix is similar matrix. According to the properties of the elementary transformation of matrix, similar matrices will become the same matrix after elementary transformation, and so their Code values are the same, and the mark the isomorphism class is the only.

#### The necessity

If the following conditions are met between two matrices, we can say that the two matrices are equal: the sizes (the row and column of matrix) of sub-graphs are the same; both the numbers of out-degree and that of in-degree are respectively equal; and so the matrices have the same *Code* value, and furthermore the corresponding symmetrical ternary number are the same. Under this condition, all the corresponding elements of these matrices are the same. Therefore, we can conclude that these matrices are equal and so they satisfy the requirement of uniqueness.

### Space compression

**Figure 4:**
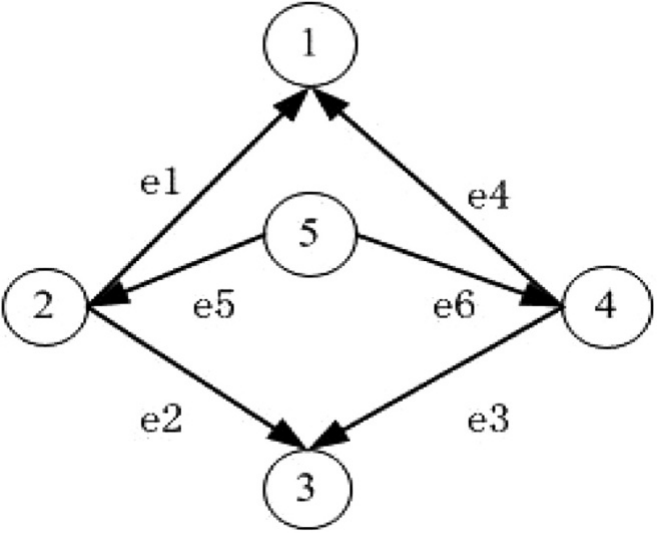
**The nodes 2 and 4 are parity nodes.** After exchanging their positions, the associated matrices remain similar and these two matrices can get into the same form by elementary transformation, thus possessing the same Code.

It appears that there is more than one parity node in a graph. The structure and function of these nodes are similar, and the topological network characteristics of these nodes are completely the same. According to the features of the parity nodes, exchanging two nodes doesn’t affect the topological structure of the whole network. This means that the corresponding associated matrices are similar after exchanging the parity nodes, as can be seen in Figure 4.

$$\begin{array}{ccccccc} e1 & e2 & e3 & e4 & e5 & e6\\ 1 & -1 & 0 & 0 & -1 & 0 & 0\\ 2 & 1 & 1 & 0 & 0 & -1 & 0\\ 3 & 0 & -1 & -1 & 0 & 0 & 0\\ 4 & 0 & 0 & 1 & 1 & 0 & -1\\ 5 & 0 & 0 & 0 & 0 & 1 & 1 \end{array}\overset{\mathrm{change}}{\Rightarrow }\begin{array}{ccccccc} e1 & e2 & e3 & e4 & e5 & e6\\ 1 & -1 & 0 & 0 & -1 & 0 & 0\\ 4 & 0 & 0 & 1 & 1 & 0 & -1\\ 3 & 0 & -1 & -1 & 0 & 0 & 0\\ 2 & 1 & 1 & 0 & 0 & -1 & 0\\ 5 & 0 & 0 & 0 & 0 & 1 & 1 \end{array}$$

We perform a pre-treatment of the searching space before enumerating the sub-graphs. In this pre-treatment, we first identify the parity nodes. In a group of parity nodes, we remove all except the node with the minimum mark number such that the network has no pairs of parity nodes, and then search directly into the network. Finally, we determine the network motifs.

**Figure 5:**
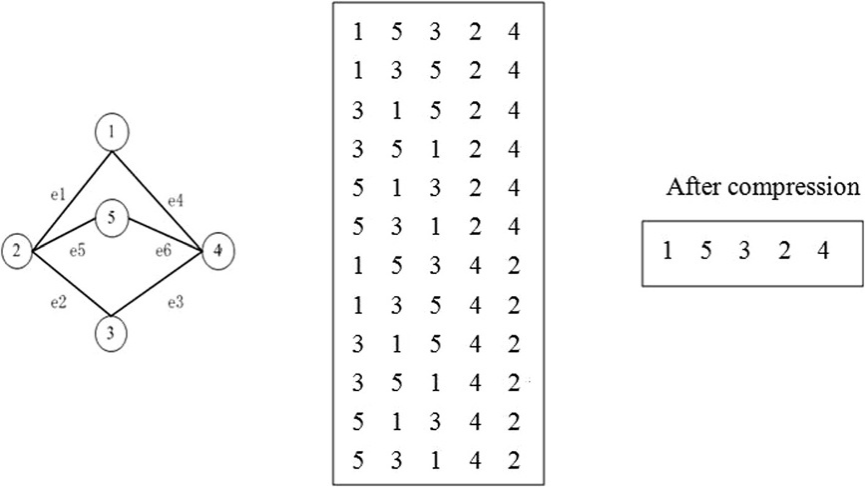
**Two groups of nodes: 2, 4 and 1, 3, 5.** The tablet is storage format of network after compression.

In Figure 5, *v*_1_, *v*_2_ represent vertex 1 and 2, respectively, and they are the remaining vertex after compression, *e*_1_ is the edge linking *v*_1_ and *v*_2_. *G*_*r*_, *G*_*p*_, *G*_*cv*_ represent the nodes and edges that remain after compression, the parity nodes that are removed and the vertex and edges connected with the parity nodes, respectively. Upon finishing the compression, we conduct a sub-graph search. At this point, we shall see that the space we need to search is considerably reduced. If we want to search a sub-graph with a specific topological structure, for instance (1,2,5), we need not go through the whole graph to find all sub-graphs with this topology, meaning that searching for sub-graphs like (2,3,5), (1,2,2), (1,4,5), (3,4,5), (1,3,4) is unnecessary. The aforementioned sub-graphs can be stored in *G*_*cv*_ and *G*_*p*_. What we need to do is replace the parity nodes correspondingly and make sure there is no repeating. In this way the searching time is reduced.

### Enumerating Sub-graphs

*Back Tracking*[24] is often used to search the optimal solutions of complex problems. It enumerates a set of partial candidates, in which we keep just the initial and the end node of a path. We also reduce the space complexity using the method of saving storage space.

We aim to enumerate all sub-graphs of a given size that occur in the real graph, and then we combine the method for enumerating all sub-graphs (*Back Tracking*) with that for compressing space. The hybrid algorithm can reduce the complexity of the sub-graphs enumeration and the searching time. The process of enumerating sub-graphs [23] can be read for reference.

## Results and discussion

In this section, we will apply our method to some real networks in order to verify the validity. We evaluate our method on the metabolic pathway of the bacteria *E. coli*, the transcription network of *Yeast*, the *Sea Urchin* network, and an electronic network. These can be freely obtained online at http://www.weizmann.ac.il/UriAlon/groupNetworksData.html[25]*.*

### Effectiveness of the algorithm

Table 1 shows the numbers and structures of motifs with different sizes observed in some different networks, and in this table, we enumerate the number of the sub-graphs that occurred in the real network and the Code that marks them with different numbers of edge in these networks.

**Table 1:** [23]**A summary of Code values and the topological structures in different networks**

***Network***	***Nodes***	***Edges***	***Size-N***	***Code***	***Motif***
*E coli*	423	519	3	8528	
			4	12925664	
				236808	
*S208*	122	189	3	5668	
			4	13698880	
				12925664	
*S420*	252	399	3	5668	
			4	12925664	
				13698880	
*S838*	512	819	3	5668	
			4	12925664	
				13698880	

In this table, the fourth column contains the numbers of motifs in the real network.

The fifth column contains the unique Code values that represent the isomorphic class, and the sixth column has the topological structures of these motifs.

In Table 1, the different numbers of edges and nodes embody the differences between networks. Most motifs are similar in the different networks, but small parts are different. If motifs have the same topological structure, their *Codes* shall be the same. This proves the accuracy of the unique mark of isomorphic sub-graphs, and also that of our algorithm. In addition, our method achieves the motif mining in different kinds of networks, and it proves that our method is both valid and extensively applicable.

### The results of compression

In order to judge the performance of our method, we carry out a series of experiments on a set of testing data. The performance of the method is evaluated by the consumption time. We enumerate all sizes of sub-graphs for testing, and then compare them with method [26]. There are no parity nodes in the electronic circuit network, so the searching time remains unchanged. However, we can compress the searching space in the network based on the properties of parity nodes.

In a network, the searching time is different when the size of the sub-graph is different. For instance as can be seen in Table 2, the S208 network costs 0.001 seconds when there are three nodes in the sub-graph, and 2.9 seconds when there are eight nodes. With increasing size, the searching time is amplified tenfold. It shows that the searching time is directly proportional to the size of the sub-graph. We will now compare the results displayed in Table 3.

**Table 2:** **List of searching times for different networks before compression**

***Time(s)***	***Size 3***	***Size 4***	***Size 5***	***Size 6***	***Size7***	***Size 8***	***N(edges) before compression***	***N(nodes) before compression***
***Networks***								
Yeast	0.013	1.860	31.281				1079	688
E coli	0.031	0.469	8.844				519	423
S208	0.001	0.002	0.031	0.125	0.579	2.953	189	122
S420	0.001	0.031	0.110	0.563	3.141	17.734	399	252
S838	0.063	0.110	0.500	2.843	17.204		819	512

The first column contains the names of different networks; the 2 through 8 columns contain the sizes of sub-graphs, and consumption time.

**Table 3:** **List of searching times for different networks after compression**

***Time(s)***	***Size 3***	***Size 4***	***Size 5***	***Size 6***	***Size7***	***Size 8***	***N(edges) after compression***	***N(nodes) after compression***
***Networks***								
Yeast	0.031	0.344	4.031				615	345
E coli	0.016	0.266	4.141				320	256
S208	0.001	0.002	0.031	0.125	0.579	2.953	189	122
S420	0.001	0.031	0.110	0.563	3.141	17.734	399	252
S838	0.063	0.110	0.500	2.843	17.204		819	512

The last two columns in this table contain the sizes of networks after reducing the parity nodes and the “repeated edges” connected with the parity nodes.

After compressing the searching space, we list the searching time in different networks with different sizes in Table 3. To avoid repeated searching, we only search one of the parity nodes and remove the edges connected with the with parity nodes which were abandoned. In this way we obtain a compressed network; the random network shall be compressed in the same way, which ensures that the random network can be used as a reference. This approach also ensures consistency of a sub-graph regardless of whether it is a network motif.

By comparing Table 3 with Table 2, it can be seen that there are no parity nodes in the electronic circuit networks, such as S208, S420, or S838. Therefore, the time for searching sub-graphs with different nodes is the same before and after compression. The subtle gaps between the results and the original results can be neglected. As for the biological networks with parity nodes, however, the more parity nodes that exist the more obvious the effect of compression is. In the example of the yeast network, the number of the parity nodes is 343, half the number of total nodes. The time for searching sub-graphs is greatly reduced after compression. With increasing numbers of sub-graphs, the efficiency of searching increases up to 10 times for 4-node sub-graphs. Of course, the efficiency of searching depends on the size of the network as well as the size of the sub-graph searched for, and the current hardware environment. In any network, the searching time is different when the size of sub-graph is different.

Through comparison of Tables 2 and 3, we confirm that the method of compressing the searching space based on the property of parity nodes is feasible, and the efficiency of searching is greatly improved by saving storage space. The advantage is particularly evident for a network with many parity nodes. This is a new attempt that ensures the identification of network motifs correctly while also improving the efficiency of searching.

### Comparison of searching times

**Figure 6:**
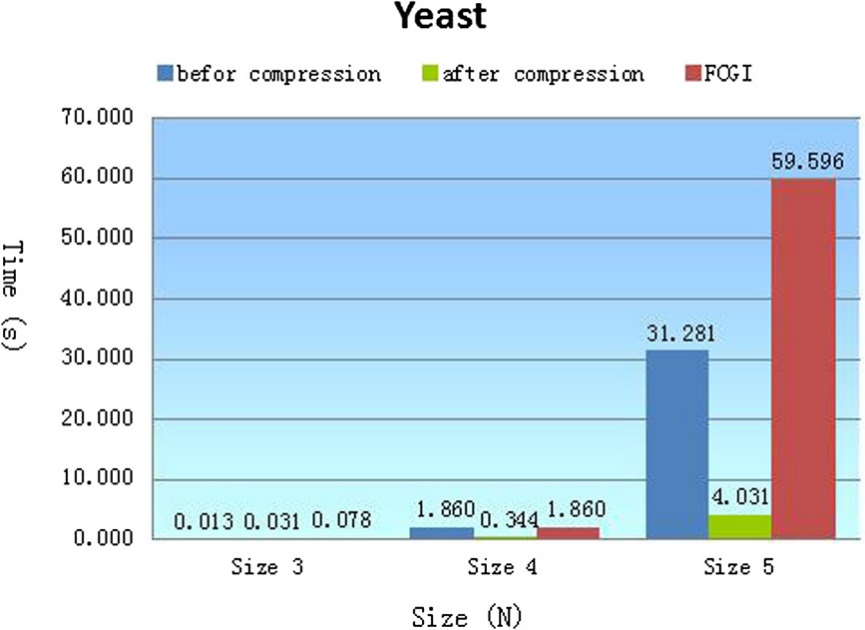
**Comparison of results from different algorithms.** The height of the blue cuboid is the consumption time before compressing the network, while the green cuboid is the consumption time after compressing the network. They are compared with the red cuboid (Rand-ESU in paper [26]). The x-coordinate denotes the size of sub-graph, the y-coordinate denotes the searching time.

In the previous sections, we have confirmed the effectiveness, accuracy and extensive applicability of our method. We have also confirmed the smaller search space and storage space obtained through the compression of parity nodes. In this section, we will compare the results obtained with our algorithm with those from alternative methods [5],[7],[17],[26]. Due to the limitations of the Feature compression for clustering graphs (FCGI) algorithm [26], we only tested and compared the Yeast network, and due to the limitations of network data just the results obtained for the E. coli network were compared to the other algorithms [5],[7],[17]. The comparison results are displayed in Figure 6 and Table 4.

**Table 4:** **Computational cost for different algorithms on the E. coli network**

***Time(s)***	***Size 3***	***Size 4***	***Size 5***	***Size 6***	***Size 7***	***Size 8***
***Algorithms***						
Before compression	0.031	0.469	8.844			
After compression	0.016	0.266	4.141			
Kavosh	0.300	1.840	14.910	141.98	1374.01	13173.74
Fanmod	0.810	2.530	15.710	132.24	1205.97	9256.61
Mfinder	31.000	297.000	23671.80			

Rows indicate different sizes of sub-graph and columns are related to different algorithms, times are in seconds.

The comparison between our method and the FCGI method was performed on the same computer environment: Intel (R)Core(TM) i3 CPU, 2.53 GHZ work station, 1.8 G RAM, Windows XP operating system, and Visual C++ 6.0. This ensures that the comparison is meaningful.

In Figure 6, algorithm performance is evaluated with the consumption time, using the yeast network as a testing data. Searching for sub-graphs in a network is the most important part of the motif mining process. The efficiency of sub-graph enumeration is, therefore, an important standard with which to measure the quality of an algorithm. In reference [26], the sub-graphs are enumerated by the *Rand-ESU* algorithm. When there are only three and four nodes, there is only a subtle difference between the time spent on enumerating sub-graphs by *Back Tracking* algorithm compared with *Rand-ESU*; this gap is negligible and can be ignored. However, with more than five nodes, the time spent even before compressing the searching space is considerably shorter than that by *Rand-ESU*, with an improvement of 27 seconds. Most striking is that after compressing the searching space the time needed is a lot shorter than *Rand-ESU*, with an improvement of 15 times (our algorithm’s consumption time is about one twelfth of the time in *Rand-ESU*).

The algorithm of Mfinder can only search a small range of sizes, and its computational cost is the highest. The FANMOD algorithm, able to find sub-graphs and isomorphic groups of sizes up to eight, results in the identification of the same numbers as Kavosh. The searching time of the Kavosh is less than that of FANMOD. With our algorithm, while the size of sub-graphs is limited to a maximum of five, the searching time is shortest.

The references [5],[7],[17], are performed on superior computer hardware than our algorithm: Intel (R)Core(TM) 2 Quad CPU, 3.2 GHZ work station, 8 G RAM. This means that the increase in speed obtained with our algorithm may be even higher.

## Conclusion

In this paper, we presented a method for mining network motifs based on space compression. All sub-graphs can be enumerated by adding edges and nodes progressively in a *Back Tracking* algorithm, which saves storage space and reduces space complexity as only the initial and end nodes of the path are counted. By taking advantages of the parity nodes, a much more efficient solution for enumerating sub-graphs during the motif mining is provided. Particularly for networks with high ratio of parity nodes, the time spent on enumerating the sub-graphs is reduced significantly compared with other common algorithms. From the results obtained, we have proved the accuracy and effectiveness of our method. By comparing the results of our method with others, we can conclude that our approach has considerably shorter searching times and more extensive applicability.

## Authors’ original submitted files for images

Below are the links to the authors’ original submitted files for images. Authors’ original file for figure 1 Authors’ original file for figure 2 Authors’ original file for figure 3 Authors’ original file for figure 4 Authors’ original file for figure 5 Authors’ original file for figure 6 Authors’ original file for figure 7 Authors’ original file for figure 8
