# Supplementary figures and images for: Motif mining based on network space compression
Source: BioData Min. 2014 Dec 11;8:29. doi: 10.1186/s13040-014-0029-x (PMC4269098; doi:10.1186/s13040-014-0029-x)

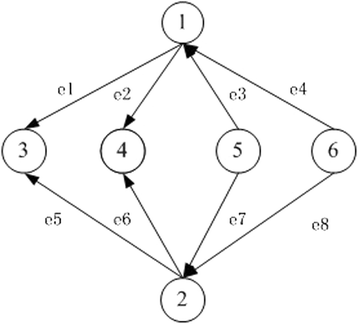

Supplement: Supplementary file 1 — Authors’ original file for figure 1 [file 13040_2014_29_MOESM1_ESM.gif]

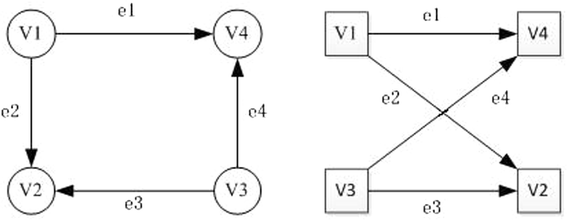

Supplement: Supplementary file 2 — Authors’ original file for figure 2 [file 13040_2014_29_MOESM2_ESM.gif]

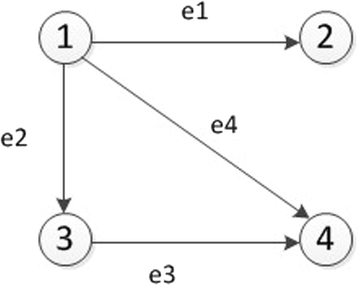

Supplement: Supplementary file 3 — Authors’ original file for figure 3 [file 13040_2014_29_MOESM3_ESM.gif]

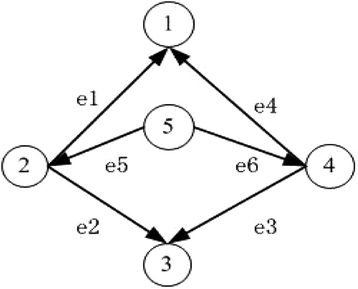

Supplement: Supplementary file 4 — Authors’ original file for figure 4 [file 13040_2014_29_MOESM4_ESM.gif]

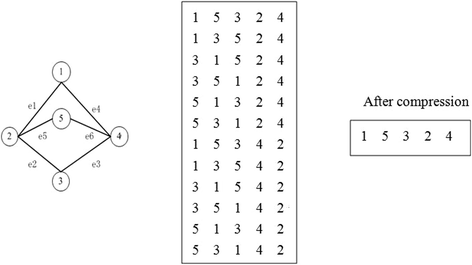

Supplement: Supplementary file 5 — Authors’ original file for figure 5 [file 13040_2014_29_MOESM5_ESM.gif]

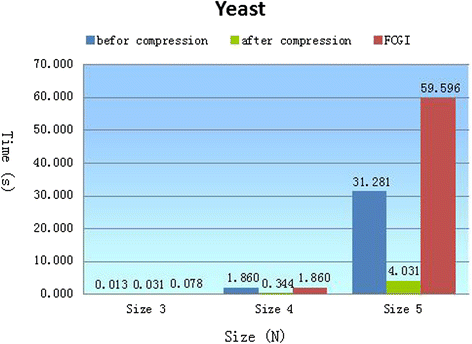

Supplement: Supplementary file 6 — Authors’ original file for figure 6 [file 13040_2014_29_MOESM6_ESM.gif]

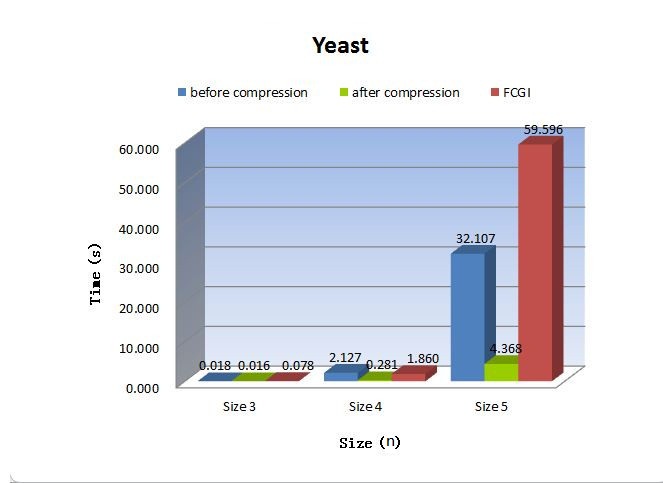

Supplement: Supplementary file 7 — Authors’ original file for figure 7 [file 13040_2014_29_MOESM7_ESM.jpeg]
